# Emergency transileocolic vein obliteration for life-threatening bleeding from gastric varices

**DOI:** 10.1016/j.radcr.2023.01.045

**Published:** 2023-02-10

**Authors:** Fumio Chikamori, Kai Mizobuchi, Ryo Hamada, Satoshi Ito, Sunao Uemura, Ryo Yamada, Hisashi Matsuoka, Nobuyuki Tanida, Niranjan Sharma

**Affiliations:** a Department of Surgery, Japanese Red Cross Kochi Hospital, 1-4-63-11 Hadaminamimachi, Kochi, 780-8562 Japan; b Department of Radiology, Japanese Red Cross Kochi Hospital, 1-4-63-11 Hadaminamimachi, Kochi, 780-8562 Japan; c Adv Train Gastroint & Organ Transp Surgery, 12 Scotland St., Dunedin, 9016, New Zealand

**Keywords:** Transileocolic vein obliteration, Splanchnic caput Medusae, Esophagogastric varices, Transjugular retrograde obliteration, Portal hypertension, Gastric variceal bleeding

## Abstract

We report a case of life-threatening bleeding from gastric varices in a patient with alcoholic cirrhosis, which was treated by emergency transileocolic vein obliteration (TIO). A 46-year-old male with a massive hematemesis was transported to our hospital by ambulance. Contrast-enhanced computed tomography demonstrated large gastric varices. Temporary hemostasis using balloon tamponade was attempted, however, bleeding could not be controlled, and his vital signs were unstable despite massive blood transfusions. First, endoscopic treatment was attempted, but the visual field could not be secured due to massive bleeding. Therefore, emergency TIO under general anesthesia was attempted. After laparotomy, 5 Fr. sheath was inserted into the ileocolic vein. Posterior and left gastric veins, which were the blood supply routes of gastric varices, were identified and embolized using microcoils and a 50% glucose solution. Hemostasis was achieved and vital signs recovered. Three days after TIO, transjugular retrograde obliteration was attempted successfully to embolize the residual gastric varices. After the procedures, his condition improved. We conclude that emergency TIO is a useful rescue option for life-threatening bleeding from gastric varices if endoscopic treatment or balloon tamponade is ineffective.

## Introduction

Advances in interventional radiology (IVR) have made elective or prophylactic treatment of gastric varices relatively easy [1], [2], [3], [4]. However, we still occasionally encounter cases of gastric variceal rupture that, although less frequent, are life-threatening mainly due to massive bleeding [5]. Endoscopic treatment using n-butyl-2-cyanoacrylate is possible only when the visual field can be secured [6,7]. When it is difficult to secure the field of view with an endoscope, balloon tamponade should be applied to control the bleeding [8]. If bleeding is still difficult to control, IVR should be applied. We report a case of life-threatening bleeding from gastric varices who has alcoholic cirrhosis. He was managed successfully by an emergency transileocolic vein obliteration (TIO) [9], [10], [11], [12].

## Case report

A 46-year-old male with hematemesis and alcoholic cirrhosis was transported to our hospital by ambulance. He had partial splenic artery embolization one year earlier for thrombocytopenia due to severe splenomegaly with a spleen volume of 762 mL. The spleen volume was reduced to 372 mL; however, he developed portal vein thrombosis as a side effect of splenic artery embolization [13,14]. He was on long-term edoxaban tosilate hydrate as an oral anticoagulant. An endoscopic examination at that time showed moderately enlarged esophageal varices and large gastric varices. He had no history of hematemesis. He was being followed up on a regular basis.

On admission, he had no jaundice. There were no features of encephalopathy. Laboratory studies revealed hemoglobin 8.5 g/dL (normal range, 13.5-17.4), total leukocyte count 16,810 /µL (3500-8000 /µL), platelet count 10.7 × 10^4^ /µL (12.3-33.1 × 10^4^ /µL), total bilirubin 1.1 mg/dL (0.3-1.3 mg/dL), albumin 3.4 g/dL (3.8-5.0 g/dL), aspartate transaminase 23 U/L (10-32 U/L), alanine transaminase 16 U/L (5-27 U/L), prothrombin time 40.7% (70%-130%), Mac-2 binding protein glycosylated isomers 3.28 COI (2+) (<1.00), and serum ammonia 154 µg/dL (12-66 µg/dL). The Child-Pugh score was 7 and the class was B. Hepatitis B surface antigen and hepatitis C virus antibody were negative.

**Fig. 1:**
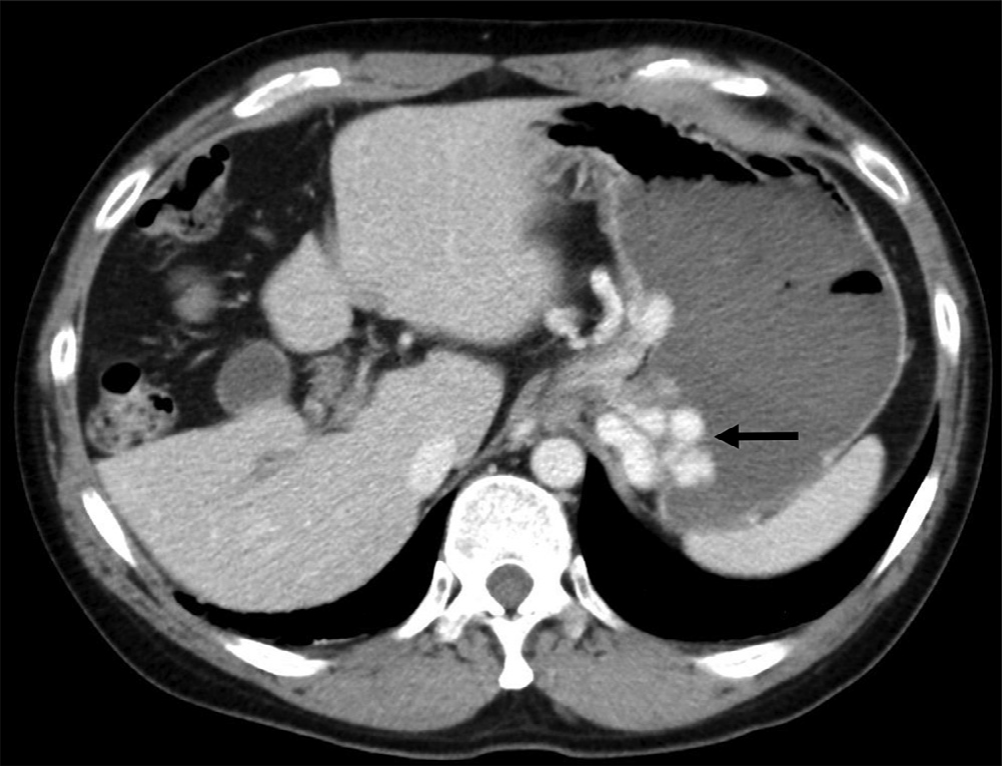
Contrast-enhanced CT before treatment shows large gastric varices (black arrow).

**Fig. 2:**
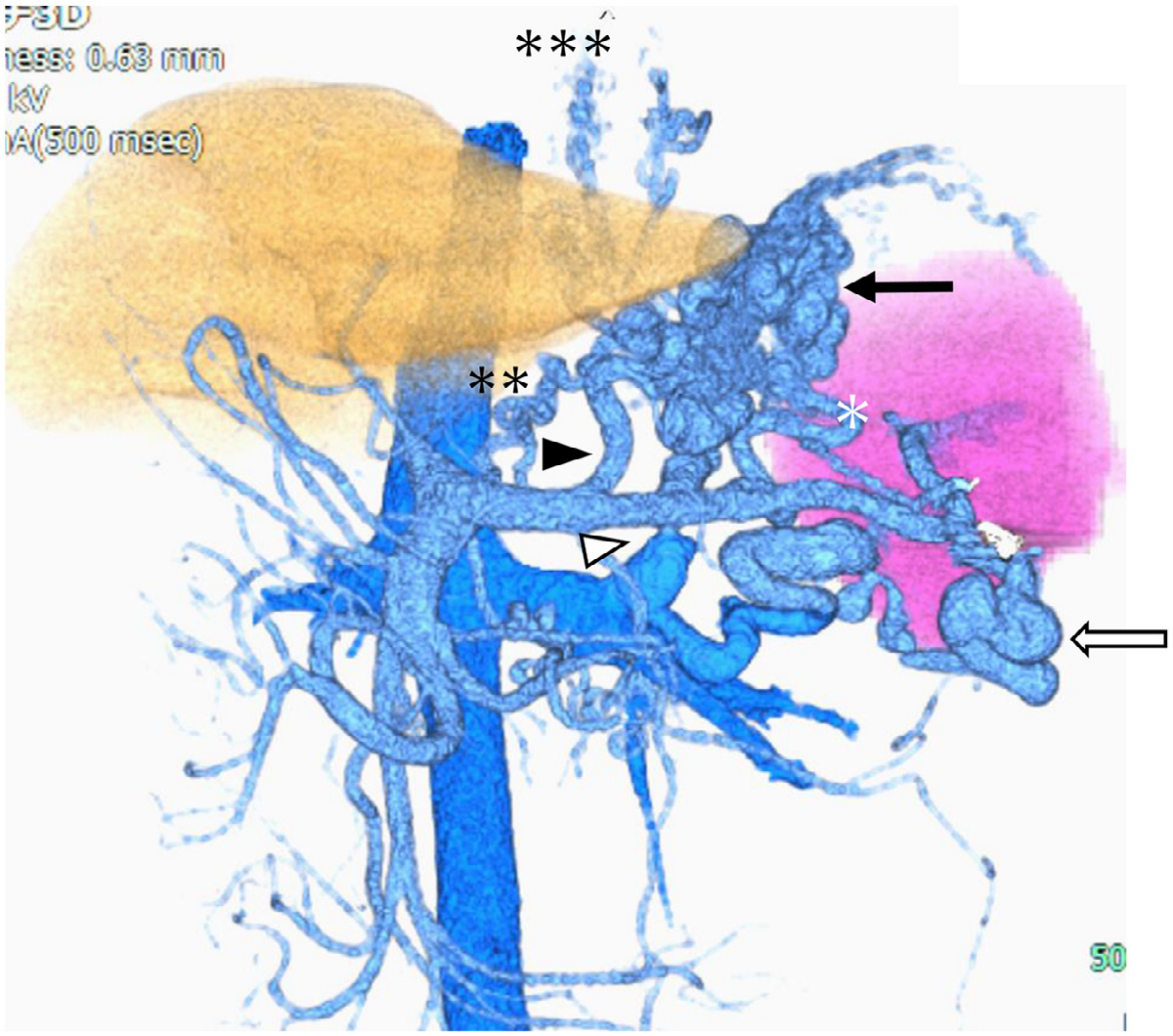
Three-dimensional CT reconstruction image before treatment shows large gastric varices (black arrow), which are supplied by the posterior (white asterisk) and left gastric (black arrowhead) veins and communicate with the left renal vein via the gastrorenal shunt (white arrowhead). Other collateral pathways are the splenorenal shunt (white arrow) and another left gastric vein (2 asterisks) communicating with the esophageal varices (3 asterisks).

Contrast-enhanced computed tomography (CE-CT) revealed gastric varices with a large gastrorenal shunt (Fig. 1). Three-dimensional CT reconstruction images demonstrated that the large gastric varices were supplied by the left and posterior gastric veins and drained into the left renal vein via a gastrorenal shunt. In addition, a splenorenal shunt was also observed as another collateral pathway. The portal vein was narrowed (Fig. 2). The spleen volume was 268 mL, and the liver volume was 776 mL; giving a spleen/liver volume ratio (S/L ratio) [15] of 0.35. This case had large gastric varices with a gastrorenal shunt despite the presence of another collateral route, the splenorenal shunt. He was considered to have severe portal hypertension.

**Fig. 3:**
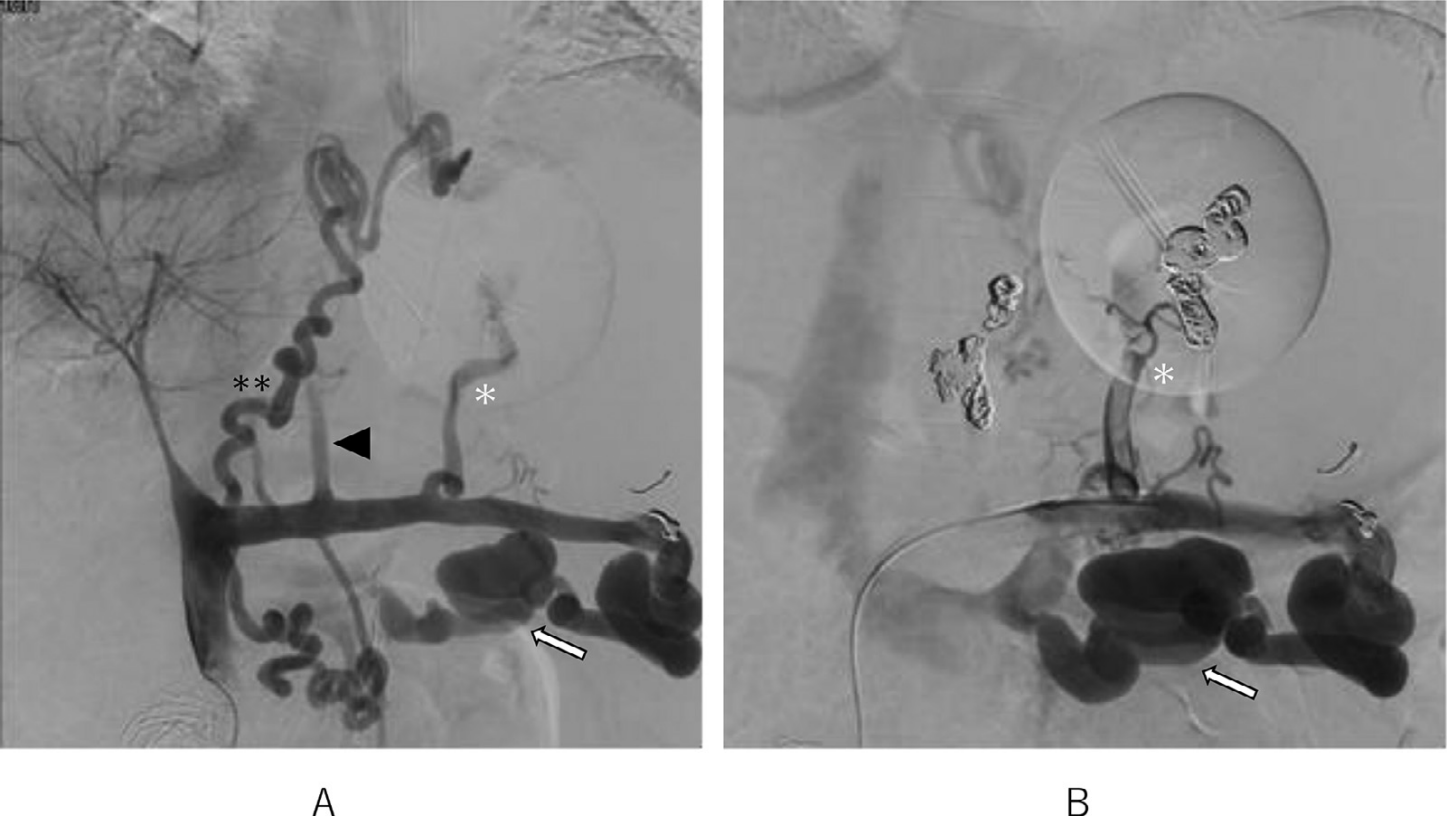
(A) Transileocolic portogram before TIO shows the posterior (white asterisk) and left gastric (black arrowhead) veins which are the blood supply routes of gastric varices. Gastric varices are not visualized because they are compressed by balloon tamponade. There is another left gastric vein (2 asterisks) that communicates with the azygos vein. The portal vein is narrowed and most of the portal vein blood flows to the splenorenal shunt (white arrow). (B) Transileocolic portogram after TIO shows a thrombus in the posterior gastric vein (white asterisk) and preserved splenorenal shunt (white arrow).

**Fig. 4:**
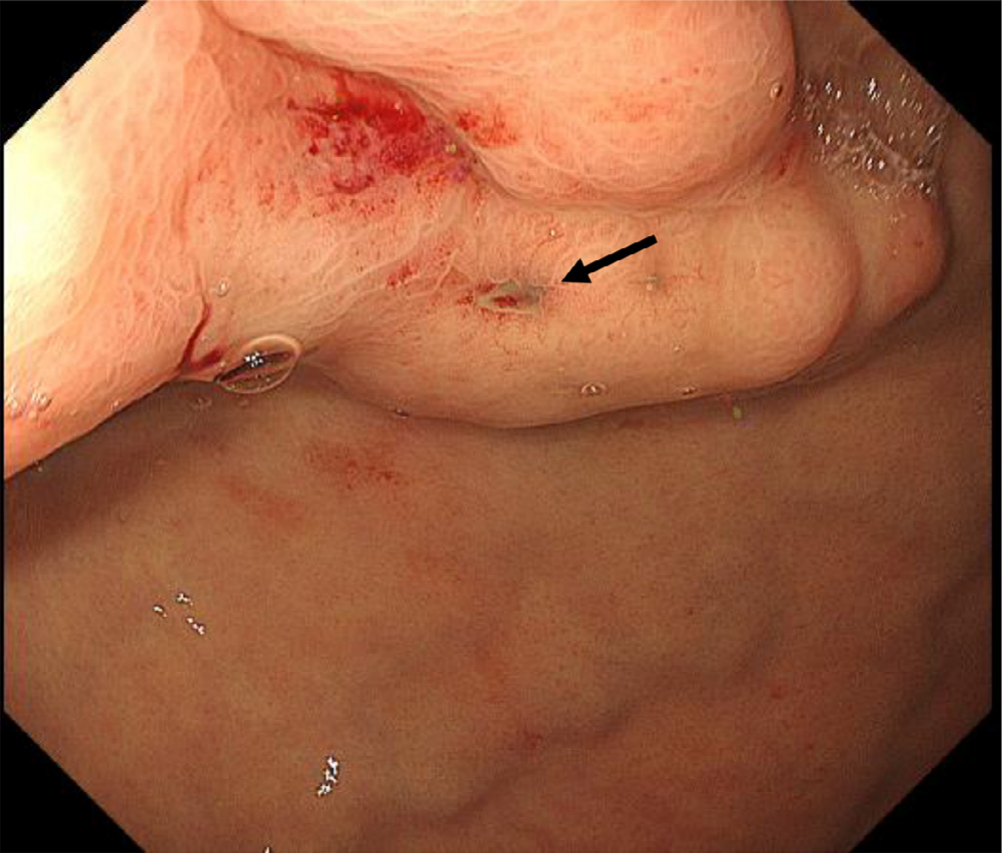
Endoscopic picture 3 days after TIO shows gastric fundal varices with a small erosion that was a bleeding point (black arrow).

**Fig. 5:**
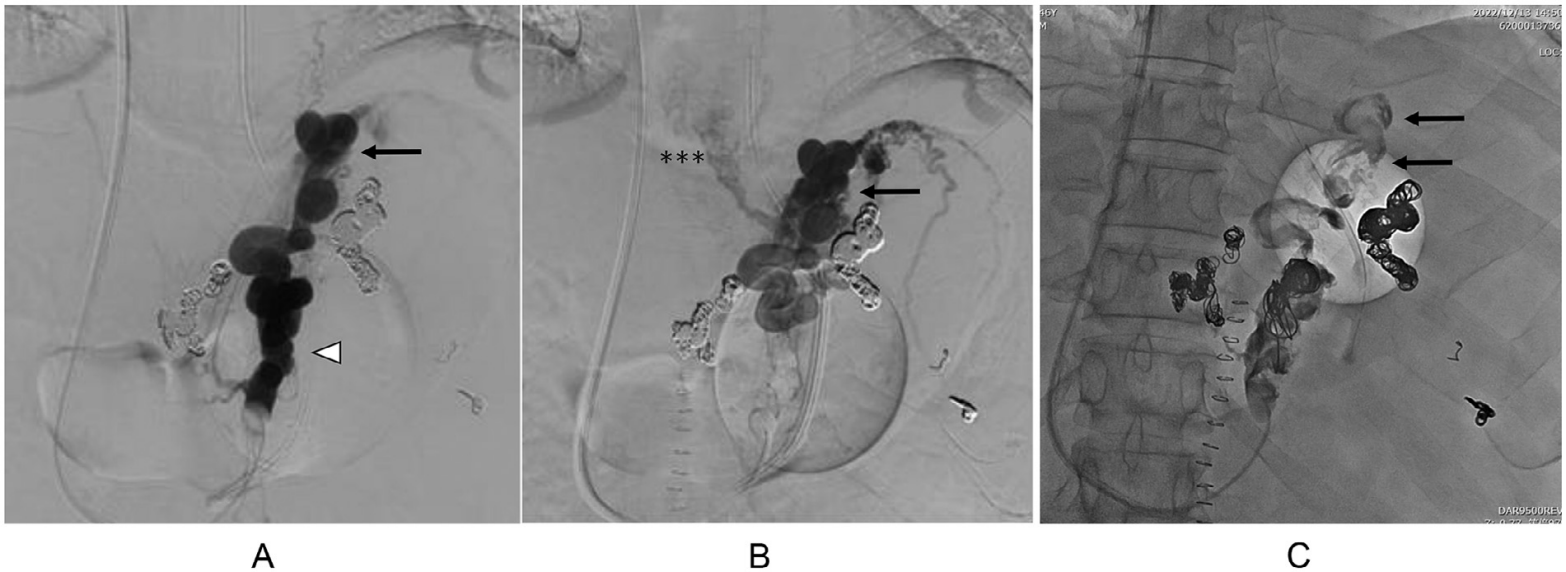
(A) Retrograde gastrorenal shunt (white arrowhead) venogram in the early phase shows residual gastric varices (black arrow). (B) Retrograde gastrorenal shunt venogram in the late phase shows residual gastric varices (black arrow), esophageal varices (3 asterisks), and short gastric veins. (C) Retrograde gastrorenal shunt venogram the day after retrograde obliteation shows the thrombus formation in the gastric varices (black arrow).

Temporary hemostasis using balloon tamponade was attempted, however, bleeding could not be controlled, and his vital signs were unstable despite massive blood transfusions. First, endoscopic treatment was attempted, however, it was impossible to secure the field of view due to massive bleeding. Therefore, emergency TIO under general anesthesia was attempted in the digital subtraction angiography room. After laparotomy, 5 Fr. sheath was inserted into the ileocolic vein. The portal venous pressure was 33.5 cmH_2_O. Posterior and left gastric veins, which were the blood supply routes of gastric varices, were identified and embolized using microcoils and 42 ml of 50% glucose (Figs. 3A and B). Temporary hemostasis was achieved. His vital signs were stabilized. Three days after TIO, CE-CT revealed that the obliteration of gastric varices was insufficient. Endoscopy revealed gastric fundal varices with a small erosion, which was the rupture point (Fig. 4). Residual gastric varices were embolized by transjugular retrograde obliteration [1,[3], [4], [5] approach. The wedged hepatic venous pressure just before transjugular retrograde obliteration was 30 cmH_2_O. Injection of 0.5-1.0 mL of absolute ethanol and 10-15 mL of 50% glucose solution into the gastrorenal shunt was carried out under fluoroscopy. This procedure was repeated at 5-minute intervals until gastric varices were clearly visualized (Figs. 5A and B). A total of 8 mL of absolute ethanol and 140 mL of 50% glucose solution were used. Additionally, 8 mL of 5% ethanolamine oleate with iopamidol was injected later into the gastric varices. The procedure was completed with the placement of the microcoils in the gastrorenal shunt. The next day, the thrombus formation in the gastric varices was verified by retrograde venography (Fig. 5C), and the catheter was removed.

**Fig. 6:**
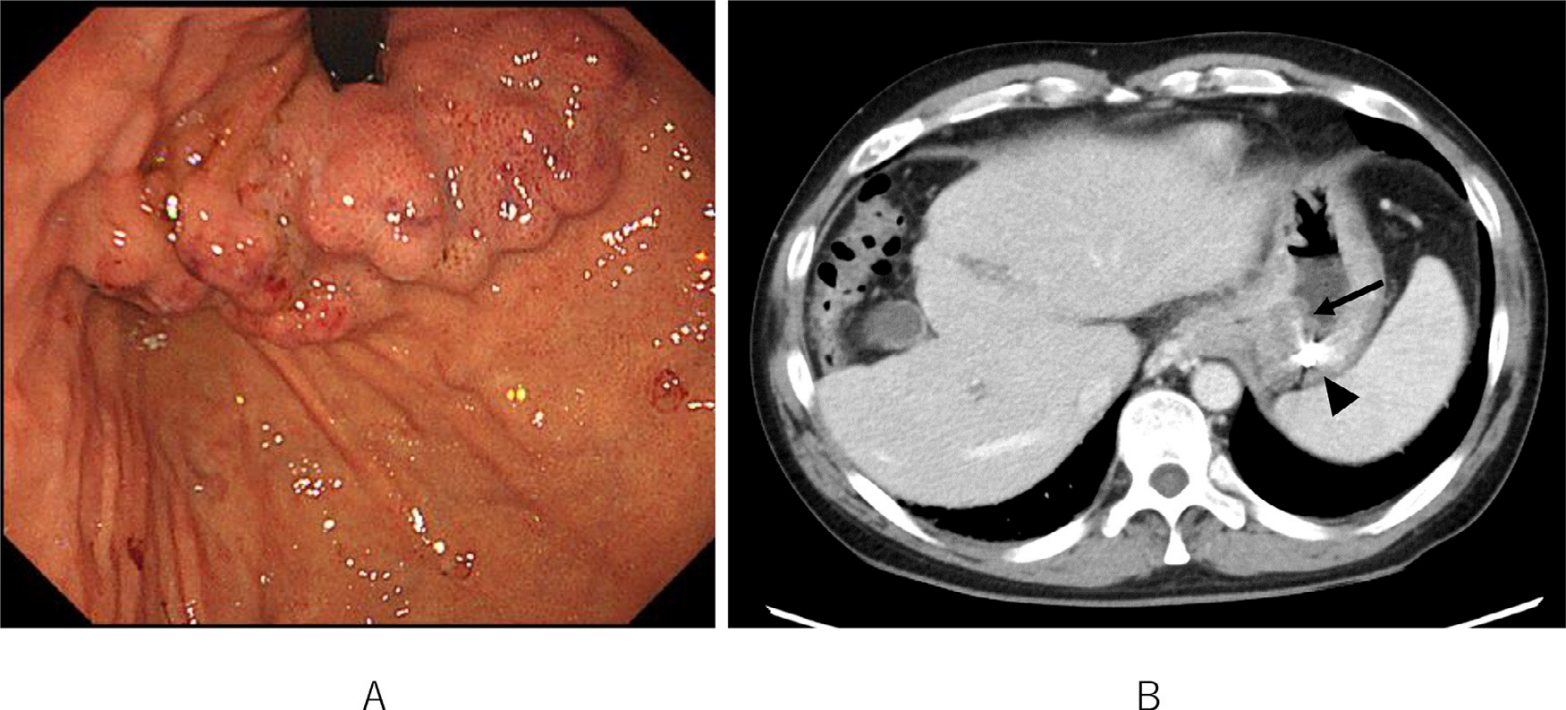
Contrast-enhanced CT and endoscopic picture after treatment. (A) Endoscopic picture 7 days after retrograde obliteration shows large gastric varices with redness which do not change the shape. (B) Contrast-enhanced CT 16 days after the procedures shows that the gastric varices are completely thrombosed (black arrow). The black arrowhead indicates the microcoils used in the TIO.

**Fig. 7:**
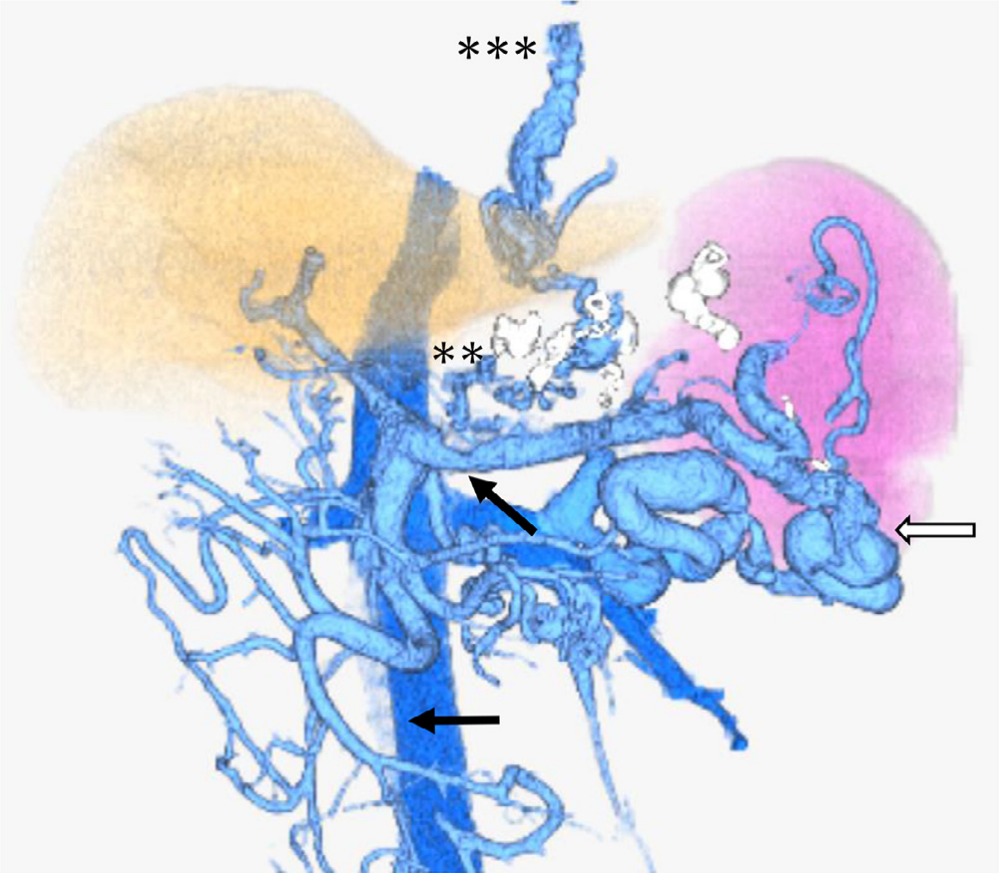
Three-dimensional CT reconstruction image after treatment. Three-dimensional CT reconstruction image shows the disappearance of blood flow in gastric varices with the gastrorenal shunt, and increased blood flow in the splenorenal shunt (white arrow). Another left gastric vein (two asterisks) is poorly embolized and communicates with esophageal varices (3 asterisks). Partial thrombosis is observed in the splenic and superior mesenteric veins (black arrow) as a side effect of IVR.

There was no clinical evidence of rebleeding. Endoscopy 7 days after the procedures showed large gastric varices with redness which did not change the shape (Fig. 6A). CE-CT on 7 and 16 days after the procedures demonstrated that the gastric varices were completely thrombosed (Fig. 6b). The splenorenal shunt was not affected. As a side effect of IVR, partial thrombosis was observed in the splenic and superior mesenteric veins. Three-dimensional CT reconstruction images showed the disappearance of blood flow in gastric varices with gastrorenal shunt and increased blood flow in splenorenal shunt and esophageal varices (Fig. 7). The initial worsening of ascites, hepatic encephalopathy, and splenic and superior mesenteric venous thrombosis gradually improved with conservative therapy, which included the administration of diuretics, branched-chain amino acids, edoxaban tosilate hydrate, and anti-thrombin III. The patient was discharged on the 23rd hospital day. A follow-up with endoscopy and CE-CT is planned in the near future.

## Discussion

We reported a case of life-threatening bleeding from gastric varices in a patient with alcoholic cirrhosis that was managed by emergency TIO [9], [10], [11], [12]. There were 3 important issues: (1) Is there an indication for prophylactic treatment for large gastric varices with portal vein stenosis? (2) What is the best strategy for the treatment of life-threatening bleeding from gastric varices? (3) Where is the position of TIO in the treatment of portal hypertension?

Large gastric varices are formed when a gastrorenal shunt protrudes into the stomach. The presence of a gastrorenal shunt usually contributes to portal pressure decompression. When esophageal varices coexist with gastrorenal shunt, they are usually small in size [16,17]. However, in this case, esophageal varices were moderately enlarged. This condition is a severe transformation in portal hypertension. Since 2020, we have proposed a new concept: “splanchnic caput Medusae” in which the spleen is her face and portal collateral pathways are her snake hairs [18]. In this case, partial splenic embolization for thrombocytopenia due to severe splenomegaly had been performed one year earlier. The Baveno Ⅶ Consensus report [8], also states that there is no indication at present for retrograde obliteration or transjugular intrahepatic portosystemic shunt (TIPS) in primary prophylaxis of gastric variceal bleeding in compensated patients. However, the patient was brought to the emergency room with hematemesis. In refractory variceal bleeding, balloon tamponade should be used as a bridge therapy to a more definite treatment [8]. Balloon tamponade is just a compression approach to the bleeding point. In this case, re-bleeding occurred when the position of the balloon deviated from the bleeding point. Treatment strategy should be determined based on portal hemodynamics and hemostatic effect. TIPS is recommended for bleeding varices that cannot be controlled endoscopically [8]. However, it was not applied due to portal vein stenosis and the presence of a large splenorenal shunt. We instituted emergency TIO as a rescue procedure.

The portal venous pressure was as high as 33.5 cmH_2_O, but the gastrorenal shunt occlusion was unavoidable because hemostasis was the top priority due to gastric variceal bleeding. There was another collateral route; splenorenal shunt. We judged that the increase in portal venous pressure caused by gastrorenal shunt occlusion could be buffered by preserving this shunt. Retrograde obliteration is indicated if bleeding from ruptured gastric varices is under control. However, in the setting of uncontrolled bleeding, retrograde obliteration carries the risk of exacerbating variceal bleeding. Therefore, it is necessary to approach the blood supply routes first and stop the bleeding, even temporarily. Percutaneous transhepatic obliteration (PTO) [19] and TIO have been reported as approaches in managing the variceal blood supply routes. Indications of PTO are limited to special cases such as refractory esophagogastric varices, ectopic varices, or chronic portosystemic encephalopathy [20]. Based upon the clinical findings this case fell within a special cases category as described above. However, PTO could not be applied due to portal vein stenosis. TIO is more invasive than PTO, but it should be selected in patients with portal vein stenosis or ascites. There are few reports of TIO [9], [10], [11], [12],21]. Yokoyama et al. [9] have summarized 15 reported cases of TIO from 1992 to 2022. In his report, the locations of varices were 1 case of esophageal varices, 3 cases of esophagogastric varices, 4 cases of gastric varices, 2 cases of duodenal varices, 1 rectal varix, 1 bile duct varices, 1 jejunal varices, and 2 anastomotic varices after abdominal surgery. Ten patients required combination therapy other than TIO, including 5 cases of endoscopic treatment, 2 cases of retrograde obliteration, 2 cases of partial splenic embolization, and 1 case of portal vein stenting. He reported that it would be difficult to embolize all collateral vessels with TIO alone.

Retrograde obliteration has a higher hemostatic effect on gastric variceal bleeding than TIPS [22,23]. We reported retrograde obliteration for gastric varices using 8Fr. cobra-shaped long sheath via jugular vein in 1996, which we called transjugular retrograde obliteration [1,[3], [4], [5]. In this case, after the improvement of vital signs, this treatment was instituted and permanent hemostasis was achieved. Although complications such as splenic and superior mesenteric venous thrombosis, ascites, and encephalopathy occurred after gastrorenal shunt occlusion, they were managed conservatively.

Severe gastric varices with massive bleeding that cannot be controlled by balloon tamponade are likely to be fatal and should be treated intensively. We conclude that TIO is one of the useful rescue procedures for life-threatening bleeding from severe gastric varices.

## Patient consent

Written informed consent was obtained from the patient for publication of this case report and accompanying images.
